# Time-Trends in Case-Fatality After Acute Myocardial Infarction Among Middle-Aged Lithuanian Adults, 2000–2023: Data from the Population-Based Kaunas Ischemic Heart Disease Register

**DOI:** 10.3390/medicina62050902

**Published:** 2026-05-07

**Authors:** Ricardas Radisauskas, Lolita Sileikiene, Dalia Luksiene, Abdonas Tamosiunas, Erika Jasukaitiene, Sarunas Augustis, Daina Kranciukaite-Butylkiniene

**Affiliations:** 1 Institute of Cardiology, Medical Academy, Lithuanian University of Health Sciences, LT-50162 Kaunas, Lithuania; lolita.sileikiene@lsmu.lt (L.S.); dalia.luksiene@lsmu.lt (D.L.); abdonas.tamosiunas@lsmu.lt (A.T.); erika.jasukaitiene@lsmu.lt (E.J.); sarunas.augustis@lsmu.lt (S.A.); daina.butylkiniene@lsmu.lt (D.K.-B.); 2 Department of Environmental and Occupational Medicine, Medical Academy, Lithuanian University of Health Sciences, LT-47181 Kaunas, Lithuania; 3 Department of Preventive Medicine, Medical Academy, Lithuanian University of Health Sciences, LT-47181 Kaunas, Lithuania; 4 Department of Internal Medicine, Medical Academy, Lithuanian University of Health Sciences, LT-47144 Kaunas, Lithuania; 5 Department of Family Medicine, Medical Academy, Lithuanian University of Health Sciences, LT-50161 Kaunas, Lithuania

**Keywords:** acute myocardial infarction, 28-day case-fatality rates, trends, sex, age

## Abstract

*Background and Objectives*. Over the past two decades, 28-day acute myocardial infarction (AMI) case-fatality rates have declined globally due to improved treatment and secondary prevention. This study aimed to evaluate AMI case-fatality levels and trends in the Kaunas population aged 25–64 from 2000 to 2023. *Materials and Methods*. Data were obtained from the population-based Kaunas Ischemic Heart Disease Registry, operating under the WHO MONICA standards. The study included adults with AMI or coronary heart disease death registered within 28 days from the onset of AMI. Case-fatality was defined as the proportion of AMI deaths among all AMI cases. Trends were assessed using JoinPoint regression. *Results*. From 2000 to 2023, 28-day AMI case-fatality in males showed no significant change, while in females it increased by 2.5% per year (*p* = 0.002). In-hospital AMI case-fatality remained stable in both sexes. Males had higher average AMI case-fatality rates than females in both age groups (25–54 and 55–64). Only females aged 55–64 showed a significant rise in AMI case-fatality (3.0%/year, *p* = 0.002). A trend change point was identified in 2006 for males (no significant trend) and in 2010 for females, after which the AMI case-fatality rates increased. Among males, in-hospital AMI case-fatality decreased significantly from 2015 to 2023 (14.7%/year). *Conclusions*. Over the two decades, 28-day AMI case-fatality among 25–64-year-old-persons remained unchanged in males but increased in females. In-hospital AMI case-fatality showed no major change in either sex. Males consistently had higher AMI case-fatality rates with age. While aging did not affect AMI case-fatality trends in males, rates rose significantly among older females.

## 1. Introduction

Since the mid-1990s, acute myocardial infarction (AMI) mortality and 28- to 30-day case-fatality rates have declined significantly among both middle-aged and older populations [1,2,3]. While AMI case-fatality rates in males are generally higher, particularly in older age groups [4], younger females have historically shown higher rates than their male counterparts. However, this sex-based gap in younger individuals has narrowed recently [5,6].

Over the last two decades, Nordic European countries have seen annual decreases in AMI case-fatality rates of approximately 3% for middle-aged males and 3.3% for females [3]. Although these AMI case-fatality rates were initially higher among those under 55 in the early 2000s, the disparity has since diminished [3].

Research from Sweden (1987–2010) indicates that AMI case-fatality rates were significantly higher among middle-aged females (35–54 years) than males; however, this disparity diminished after adjusting for comorbidities [7]. Among older populations, AMI case-fatality rates continue to decline, with annual reductions of 1.2–3.3% for males and 3.3–3.9% for females in countries like Lithuania and Germany due to improved treatment. Despite these improvements, overall, AMI case-fatality remains lower in females than in males [8]. In older cohorts (75–84 years), significant downward trends are evident: Germany reported annual decreases of 3% for males and 10% for females [9]. While similar declines are seen in Norway and Finland, inpatient fatality for females over 75 in certain regions remains comparable to or slightly exceeds that of males [3,5,10].

Significant sex-based disparities in AMI case-fatality among young adults (under 55) have diminished over time, narrowing from a twofold difference in the mid-1990s to approximately 1.3 times more recently [3,8]. This trend is driven by a more rapid decline in absolute case-fatality among females, which has reduced the gap between younger and middle-aged cohorts—though older males still frequently exhibit slightly lower long-term survival rates than females [3]. These improvements are largely attributed to advancements in clinical management, including higher rates of percutaneous coronary intervention (PCI), revascularization, and the widespread use of anticoagulants, beta-blockers, and statins [11,12,13,14,15,16]. Additionally, enhanced primary and secondary prevention, especially among females, alongside increased adherence to clinical guidelines and increased awareness has played a key role [17]. However, younger females often still experience treatment delays due to atypical symptoms, and regional variations in hospital care persist [18].

Currently, the analysis and assessment of long-term case-fatalities in AMI, including the COVID-19 pandemic period, is insufficient in the context of Eastern Europe and the Baltic countries, and the studies that have been conducted so far are quite contradictory and require more detailed insights in terms of both rates and its trends. The implementation and evaluation of the effectiveness of cardiovascular disease prevention programs in the Eastern European and Baltic region also requires research in this context.

Study aim—to determine and evaluate 28-day AMI case-fatality rates and trends among the Kaunas 25–64-year-old-persons from 2000 to 2023 according to the population-based Kaunas (Lithuania) Ischemic Heart Disease (IHD) Register.

## 2. Materials and Methods

### 2.1. Study Sample

This study analyzes data from the Kaunas IHD Registry spanning the period 2000–2023. Established in 1983 based on the World Health Organization (WHO) recommendations and the MONICA project framework, the registry monitors an annual population of approximately 200,000 residents aged 25–64 in Kaunas, Lithuania. It captures all cases of AMI and coronary-related deaths within this demographic. Detailed descriptions of the primary data sources are available in the previous literature [19].

### 2.2. Data Definitions

Study data were assessed based on final clinical diagnoses of AMI events, categorized using ICD-10 codes (I21–I22 for acute myocardial infarction and I20.0 for unstable angina pectoris) alongside epidemiological diagnostic categories (EDC) for “definite” and “possible” AMI.

Following the WHO MONICA protocols, “definite” AMI for non-fatal cases was identified by definite or possible clinical presentations, definite elevation of cardiac enzymes, and having definite or possible ischemic electrocardiographic (ECG) changes.

For non-fatal cases, “possible” AMI was identified by specific clinical presentations, definite clinical findings, cardiac enzyme levels indicating possible elevation, and possible ischemic ECG changes.

For fatal cases, this classification relied on clinical investigations, autopsy results (e.g., >50% coronary stenosis or previous AMI scarring), and medical history of IHD in the outpatient documents [19].

Mortality data were sourced from the Kaunas Civil Registry Office and the Lithuanian Cause of Death Register [20], with all medical death certificates reviewed and coded according to ICD-10 standards [21].

Fatal cases diagnosed with codes I20–I25 were selected and verified. To ensure comprehensive identification of IHD-related mortality according to the WHO MONICA guidelines, additional ICD-10 codes were screened, including arterial hypertension (I10–I15), other heart diseases (I30–I52), cerebrovascular diseases (I60–I69), diseases of arteries, arterioles, and capillaries (I70–I77), and metabolic disorders, such as diabetes, obesity, and dyslipidemia (E10–E14, E65–E68, E78).

The analysis focused on the individuals who died within 28 days of AMI onset, regardless of the location (in-hospital or out-of-hospital). Cases classified under EDCs as “definite” AMI, “possible” coronary death, or “insufficient data” to confirm or exclude IHD, were included.

Data consistency and comparability were maintained through standardized detection methods and diagnostic criteria throughout the study period, with results analyzed by sex and age.

### 2.3. Statistical Analysis

The 28-day AMI case-fatality rates (in percentage) were calculated as a proportion of deaths to all AMI events multiplied by 100. The 28-day case-fatality rates from AMI were directly adjusted within 10-year age groups to the World Standard Population [22].

To assess temporal trends and their changes, JoinPoint regression analysis was performed using the JoinPoint software Version 5.4.0.0 (Statistical Research and Application Branch, National Cancer Institute, Bethesda, Maryland) [23]. This method identifies calendar years in which statistically significant changes in trends occur (joinpoints) and estimates annual percentage changes (APCs) for each time segment, as well as the average annual percentage change (AAPC) with 95% confidence intervals (CIs) over the entire study period from 2000 to 2023.

Given the relatively long time series (24 annual observations), the maximum number of joinpoints was set to four, in accordance with the recommendations of the JoinPoint Regression Program, to ensure sufficient model flexibility while avoiding overfitting.

Model selection was based on the permutation test implemented in the JoinPoint software, which sequentially evaluates models with increasing numbers of joinpoints (from 0 up to the pre-specified maximum) and selects the most parsimonious model that provides a statistically significant improvement in model fit.

The analysis started with a zero-joinpoint model (i.e., a single log-linear trend over the entire study period). Additional joinpoints were included only if the permutation test indicated a statistically significant improvement in fit at a two-sided *p*-value < 0.05.

Consequently, a zero-joinpoint model was retained in instances where no statistically significant changes in trend slopes were detected, representing a stable long-term trend without identifiable breakpoints.

The methodology of JoinPoint regression is based on fitting a series of connected linear regression segments on a logarithmic scale and identifying statistically significant changes in slope at joinpoints [24].

In our analysis, we focused primarily on the AAPC estimates for the entire study period. This approach was chosen because analyses of shorter time segments may be unstable and prone to bias due to small absolute numbers, particularly in age- and sex-specific subgroups, such as younger females. Reporting AAPC therefore provides a more robust and interpretable summary measure of long-term trends in AMI case-fatality rates.

### 2.4. Ethical Approval

Before the analysis, all patient records/information were anonymized and depersonalized. The study was approved by the Lithuanian Bioethics Committee (No. 14-27/03, 3 December 2001) and the Kaunas Regional Biomedical Research Ethics Committee (No. BE-2-39/19, 19 April 2021).

## 3. Results

Over 24 years (2000–2023), a total of 10,912 AMI cases were recorded, of which 3060 (28.0%) did not survive 28 days from the onset of the disease. It was evaluated that among the AMI patients who did not survive 28 days, 2535 (30.3%) were males and 525 (20.6%) were females (*p* < 0.0001).

**Figure 1:**
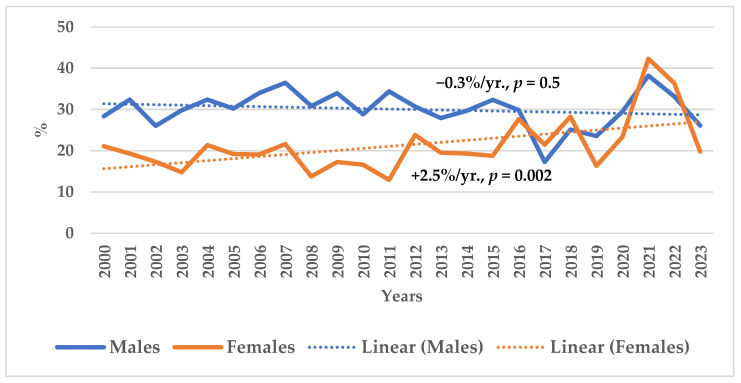
The trends of 28-day acute myocardial infarction case-fatality rates in Lithuanian urban (Kaunas) residents aged 25–64 years by sex during 2000–2023.

An assessment of 28-day AMI case-fatality rates among the middle-aged population (25–64 years) of Kaunas, Lithuania, from 2000 to 2023 revealed divergent trends between the sexes. While male AMI case-fatality rates remained largely stable throughout this period, female AMI case-fatality rates showed a significant upward trend (Figure 1).

Between 2000 and 2023, 20.3% of AMI deaths occurred in hospitals (*n* = 620). Among these, males accounted for a lower proportion (19.0%) compared to females (26.3%) (*p* < 0.001). In contrast, out-of-hospital deaths among individuals aged 25–64 were more common in males (81.0%) than in females (73.7%) (*p* < 0.0001).

**Figure 2:**
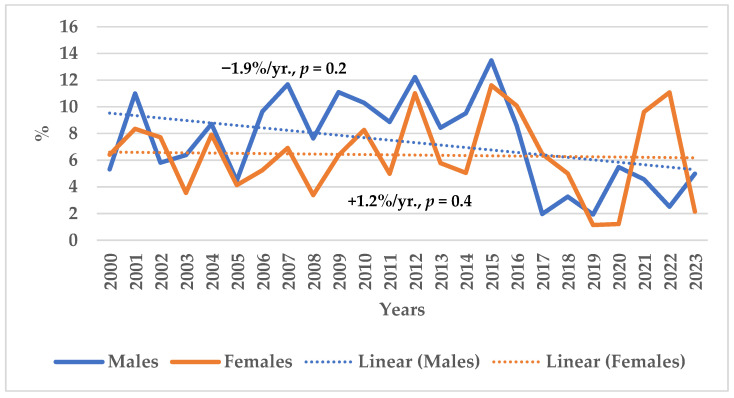
The trends of in-hospital 28-day acute myocardial infarction case-fatality rates in Lithuanian urban (Kaunas) residents aged 25–64 years by sex during 2000–2023.

Over the same period, in-hospital case-fatality rates for AMI in Kaunas residents aged 25–64 showed no meaningful change across sexes (Figure 2).

Table 1 shows 28-day AMI case-fatality rates and trends (2000–2023) among individuals aged 25–64 in Kaunas by sex and age.

Overall, males had significantly higher AMI case-fatality rates than females in both age groups, 25–54 and 55–64 years. Among those aged 25–54, the average rate was 23.55% for males vs. 16.77% for females, while in the 55–64 group it was 34.28% for males vs. 23.34% for females (*p* < 0.05) (Table 1).

The 28-day AMI case-fatality rates fluctuated widely over time. Trend analysis showed no significant changes over time for males in either age group or for females aged 25–54. However, females aged 55–64 experienced a significant increase in AMI case-fatality rates (Table 1).

Table 2 summarizes 28-day in-hospital AMI case-fatality rates and trends (2000–2023) among residents aged 25–64 in Kaunas by sex and age groups (25–54 and 55–64). Overall, no significant differences were observed between males and females in either age group. In-hospital AMI case-fatality rates fluctuated considerably over time. A pronounced variability in AMI case-fatality rates was found in the group of females aged 25–54. Very low AMI case-fatality rates were found in females aged 25–54 in 2004, 2012–2014, and during the COVID-19 pandemic period. In-hospital AMI case-fatality rates for Kaunas males and females in the aged groups of 25–54 and 55–64 over the past two decades were without significant changes (Table 2).

Table 3 presents trends in 28-day AMI case-fatality rates by sex and age. For males aged 25–64, a change point was identified in 2006, but no significant trend changes were observed before or after this year. In contrast, for females aged 25–64, 2010 marked a key point: the rates remained stable from 2000 to2010, but increased significantly from 2010 to 2023 (Table 3).

Among younger individuals (25–54 years), 2021 was identified as a change point for both sexes, yet no significant trends were detected. For the older group (55–64 years), trends differed by sex. In males, a 2019 change point was found, but no significant changes occurred in either period. In females, 2009 was a key point: rates were stable from 2000 to 2009 but rose significantly from 2009 to 2023 (Table 3).

Table 4 presents trends in-hospital AMI case-fatality rates by sex and age. For males aged 25–64, a change point was identified in 2015. While no significant changes were observed before this year, in-hospital AMI case-fatality rates decreased significantly from 2015 to 2023. In females aged 25–64, a change point occurred in 2003, but no significant trends were found in either earlier or later periods.

Among younger individuals (25–54 years), change points were detected (2007 for males and 2021 for females), but no significant changes in trends were observed. For the older group (55–64 years), patterns differed by sex. In males, 2015 marked a key point: rates were stable before this year but declined significantly afterward. In females, a 2021 change point was identified, yet no significant changes were observed in either time (Table 4).

## 4. Discussion

Our study found that over the past two decades, significantly more males than females died within 28 days of AMI (one-third of males and one-fifth of females). While more males than females died in the pre-hospital period, on the other hand, more females than males died in the hospital within 28 days (more than a quarter of females and one-fifth of males).

Between 2000 and 2023 in Kaunas, overall, AMI case-fatality rates remained stable in males but increased significantly in females, particularly among those aged 55–64. Although males consistently had higher average case-fatality rates than females in both age groups, no significant long-term changes were observed for males or for females aged 25–54; the increase was confined to older females.

Trend analysis identified several change points, but most did not correspond to meaningful shifts. For males, points such as 2006 (overall) and 2019 (ages 55–64) showed no significant changes. For females, 2010 (ages 25–64) and 2009 (ages 55–64) marked the beginning of significant upward trends, while younger women (25–54) showed no meaningful changes despite a 2021 breakpoint.

Sex differences in AMI and fatal outcomes are well documented [18]. These differences are likely due to the interaction of diverse and complex biological sex differences and clinical features rather than to a single cause. From a biological standpoint, females and males show important differences in coronary artery structure and disease processes. Females more frequently experience microvascular dysfunction and plaque erosion rather than plaque rupture. Their coronary arteries are generally smaller, which can make medical procedures more challenging, and they more often present with non-obstructive coronary artery disease. Because of these factors, imaging tests like angiographies may appear less definitive in females, increasing the likelihood of missed diagnoses and, as a result, less effective treatment during AMI. Hormonal influences also play a significant role. Estrogen provides protective effects on blood vessels by reducing inflammation and promoting dilation. However, this protection declines after menopause, when endothelial function worsens and the risk of blood clot formation increases. This helps explain why younger females tend to have lower rates of heart disease, while older females—especially after menopause—experience worse outcomes following AMI [25,26,27].

When evaluating the epidemiological aspects of sex differences in AMI, it should be noted that females have a higher short-term (in-hospital) mortality rate than males [18]. A study conducted in Italy found a twofold higher in-hospital mortality rate in women compared to men, which was 10% and 5%, respectively, and this difference was found in a younger female population (<55 years) [28].

In contrast, in-hospital AMI case-fatality rates remained largely stable for both sexes over the study period, with no significant differences between males and females across age groups. However, improvements were observed in males after 2015: in-hospital AMI fatality rates declined significantly, especially among those aged 55–64. No comparable significant changes were detected in females, whose rates remained stable despite identified breakpoints (e.g., 2003 and 2021). Overall, AMI fatality trends worsened only in females—especially older females—while in-hospital outcomes improved mainly in males and remained unchanged in females. It should be emphasized that the fatality rate from AMI among the young females studied (aged 25–54) was very low in some years, especially during the COVID-19 pandemic, which may have skewed the study results.

Other population-based studies in Europe, of which there were few, reported sex- and age-specific mortality trends, including deaths occurring before hospital admission and deaths within the first 28 or 30 days after hospital admission [4,29]. Studies in France [30] (2006–2014), England [29] (2002–2010), and Spain [4] (1996–2008) found a significant reduction in mortality from AMI among older males (65–84 years) but also decreased among younger males (<65 years). In the KORA MI registry study (Germany), among persons aged 25–54 and 55–64 years, no changes were observed in the AMI case-fatality rate during the first 28 days from 2004 to 2015 [9]. In a study in Italy (2009–2018), both males and females had a decreasing mortality rate from AMI. Still, in the age group up to 75 years, the case-fatality rates from AMI were lower in females, whereas in persons > 75 years, AMI case-fatality rates were higher in females than in males [31]. According to data from England from 2015 to 2018, the contribution of case fatality from AMI was largest in women aged 55–64 and 65–74 years, and in men aged 75–84 years. Pre-hospital AMI fatality rates were slightly higher in males than in females in most age groups, whereas in-hospital AMI case-fatality rates were higher in females with increasing age, including those aged 65–74 years [32].

When evaluating data from the female population, mixed trends were found. Some authors reported a significant decrease in AMI mortality in young females [29,33,34]. Other researchers in Spain and France have also found declining trends in AMI mortality among middle-aged women [4,30]. In contrast, other researchers presented generalized trends from six European countries and reported an increase in 28-day AMI case-fatality among older females (65–74 years) from 2005 to 2010 [5]. In some countries, such as Australia, Canada, New Zealand, and England, when evaluating data on case-fatality from AMI from 2002 to 2015, the highest case-fatality rates were found in England, although its decline in AMI case-fatality rate was also the greatest. The decline in case-fatality was greater than the decline in deaths among those under 55 years of age [35].

The decreasing 28-day AMI case-fatality rate may reflect the increased use of evidence-based interventional therapies in acute care settings for AMI patients and the use of drug combinations for the treatment of IHD in AMI patients, which were recommended in the 2000 guidelines for acute coronary syndromes in older patients [11,36,37]. In our study, individuals aged 55–64 years experienced the highest number of fatal AMI cases, especially in-hospital AMI deaths, and an increase in AMI cases was observed during the COVID-19 pandemic, although this is not reflected in the overall trend over two decades. Further trend analysis will help to more accurately assess the impact of the COVID-19 pandemic on AMI mortality.

Researchers found that during the COVID-19 pandemic, mortality from circulatory system diseases and mortality from cardiac pathology significantly increased, the number of catheter procedures performed decreased, and coronary care deteriorated both in hospital and at home [38,39,40,41]. In our study, when assessing in-hospital fatality after AMI during the COVID-19 pandemic, it should be emphasized that there was a significant increase in fatalities during this period in the older age group (55–64 years) of females and a very low fatality rate from AMI in the younger age group (25–54 years). This could be related to the more frequent complications of COVID-19 infection in older females and worse outcomes compared to younger women. No such trends were found in the older male population.

Since our 28-day fatality rates include both deaths occurring before hospital admission and deaths occurring within 28 days of hospital admission, changes in these rates are also influenced by other factors that are of considerable value, especially before hospital admission, such as timely diagnosis of coronary symptoms by healthcare institutions’ doctors and the quality of coronary care provided to individuals with pre-existing coronary artery disease before hospital admission [42]. When analyzing the data by sex, in our study, 28-day AMI case-fatality rates increased significantly in older females (55–64 years), although in-hospital AMI case-fatality rates did not change during the study. It remains unclear whether this reflects somewhat less progress in the treatment of AMI in females, or whether, conversely, out-of-hospital AMI case-fatality trends mask possible improvements in the quality of coronary heart care provided after hospitalization.

In recent years, much effort and funding have been invested in Lithuania to improve the management, timely and effective diagnosis, and treatment of AMI, including outpatient and inpatient care of AMI cases [43]. In Lithuania, the currently increasing rate of myocardial reperfusion, as a measure of the effectiveness of AMI treatment, is similar to the data from Western and Northern European countries [44]. All the above indicators may have contributed to the decreasing trend in AMI case-fatality rates.

It should be noted that during the study period or in separate periods, no significant trends in AMI case-fatality rates were observed in the Lithuanian middle-aged female population, and in the older age group (55–64 years), a significant increase in AMI case-fatality rates was observed. This may be related to lower access to health care services for females and shortcomings in the management of females with AMI, unclear clinical presentation and diagnosis of the disease, or other serious comorbidities, such as lung disease, diabetes, or kidney disease, among females [45,46,47]. Gender differences in clinical presentation cannot be ruled out. When assessing symptoms, women are more likely to present with fatigue, dyspnea, nausea, and back/jaw pain, but less likely to present with the classic “crushing chest pain,” which results in more frequent misdiagnosis and delayed hospital admission. Diagnostic challenges in women also require caution due to lower sensitivity of standard electrocardiographic (ECG) criteria in women (due to anatomical differences), and less frequent recognition of non-obstructive or atypical myocardial infarction. There are also differences in heart structure and electrical activity. Females typically have smaller heart chambers and a slightly different heart position, which can reduce the sensitivity of ECG tests and make it harder to detect classic signs like ST-segment elevation. Additionally, causes of death differ; females are more likely to die from mechanical complications such as cardiogenic shock or pulmonary edema, whereas males more often die from arrhythmias like ventricular fibrillation, indicating distinct underlying disease mechanisms [26,27,28]. This contributes to underdiagnosis or delayed diagnosis, resulting in poorer outcomes. The stability of AMI lethality in females can be explained by different clinical manifestations due to co-morbidities, slightly delayed access to healthcare institutions, and, consequently, some differences in AMI treatment, which potentially result in more deaths [48,49,50,51,52,53].

The development of heart failure (HF) during initial hospitalization is recognized as one of the major prognostic factors for poor outcomes in patients with AMI [54,55]. Factors contributing to the pathogenesis of HF development during AMI hospitalization include exacerbation of pre-existing HF and comorbidities, myocardial necrosis, or mechanical complications. Several studies based on administrative datasets have reported opposing trends in the incidence of HF during AMI [10,56,57].

It is probable that the proportion of HF cases diagnosed during hospitalization for AMI has increased due to the rising average age of hospitalized patients, and that the frequency of HF development during hospitalization has decreased due to the implementation of timely and effective myocardial revascularization and troponin-based AMI diagnostics in healthcare institutions, which allow for the identification of milder forms of AMI, making the risk of HF development lower. Some studies have found a smaller and decreasing proportion of AMI cases that had a HF complication, both in younger and older AMI patients, and especially in females [31]. Researchers found that patients with AMI who were diagnosed with HF had up to a 4-fold higher case-fatality rate than patients with AMI who did not have HF, including in the youngest and oldest age groups [58].

Treatment differences (healthcare system factors) have also been identified, including unequal care. Women are less likely to undergo percutaneous coronary intervention (PCI) than men due to lower use of evidence-based medications (antiplatelet drugs, statins, beta-blockers). The incidence of PCI in females is about 40%, compared to 61% in males. These differences significantly influence mortality differences [59,60].

Some Eastern European countries, such as Poland, Romania, and the Czech Republic, have made significant progress in reducing AMI mortality rates, but significant disparities remain [61]. Despite the improvement, the mortality rate in Lithuania is still quite high (up to 2-fold higher). Poland has achieved the biggest reduction in AMI mortality trends, most likely due to comprehensive prevention and treatment strategies. This improvement is associated with strengthened primary and secondary prevention implementation, wider use of statins, and prompt acute coronary care measures [62]. Romania and the Czech Republic have also made considerable progress but still face challenges in reducing AMI mortality to the levels observed in Western Europe [63].

Addressing these disparities requires continued investment in improving access to quality health care and implementing targeted interventions to reduce cardiovascular risk factors among Lithuanian females, especially in older age, when females’ hormonal protection ends.

### Study Strengths and Limitations

Our study also has some limitations. First, it is a retrospective study, in which the data collected for each AMI case depend on the accuracy of the medical records, the life and medical history data provided in the medical charts and outpatient cards, and the correctness and completeness of the clinical course data and the diagnostic procedures used, especially in cases where the studied case was very acute and ended in death. In cases of sudden death, the data were reviewed at autopsy or the clinical course data were used if the autopsy procedure was not performed. AMI cases that ended in death after hospital discharge were recorded by reviewing the National Causes of Death Registry database, including during the COVID-19 pandemic. Second, small sample sizes in some age groups often lead to unstable estimates because when there are very few individuals, even a small change can significantly change the rate. With small numbers, chance plays a larger role than significant differences. At the same time, in small subgroups, potential data collection inconsistencies have a greater proportional impact. It should be noted that the high variability in rates in small age subgroups may be related to low data reliability rather than reflecting large differences in the real world. Third, we did not compare fatal AMI cases by type of AMI (non-ST-elevation and ST-elevation), which limited our study results. The MONICA project guidelines do not provide detailed trends in the treatment aspects of AMI cases that could explain the decreasing mortality from ischemic heart disease; therefore, our results are limited. Fourth, the accuracy of the study results may have been affected by incorrect coding of death codes in patients with AMI. In our study, we could not exclude the possibility that our results did not show other increasing or decreasing trends during the study period due to incorrect coding, incorrect epidemiological classification of cases, or misdiagnoses, especially in individuals with SARS-CoV-2 infection. Similarly, the diagnosis of AMI cases may have been misleading, especially if the death occurred suddenly outside of the hospital, since autopsies are not always performed in cases of sudden death, and diagnoses are made based on previous medical records. Therefore, we cannot rule out the possibility that the death code on the death certificate may have influenced the study findings. Previous studies have shown that errors on the death certificate have led to inaccurate data that affect mortality statistics, although previous studies have shown high accuracy in coding AMI cases [64]. Finally, given that previous studies have shown that cardiovascular mortality in younger individuals may be underestimated on death certificates [65], our results may underestimate the true mortality in younger age groups. In our study, in some years, especially during the COVID-19 pandemic, the number of females hospitalized and dying from AMI in the group of young females (25–54 years) was very low, which may have been important for assessing changes in rates and their trends.

## 5. Conclusions

During 2000–2023, 28-day AMI case-fatality rates for males aged 25–64 years were without significant changes, while for females it increased. The in-hospital AMI case-fatality rates for both males and females did not change substantially during this period. The average AMI case-fatality rates for both males aged 25–54 and 55–64 were significantly higher than for females. The 28-day AMI case-fatality rates for Kaunas 25–54- and 55–64-year-old males and for females aged 25–54 years over the past two decades showed no significant changes, with some bias in females’ rates; however, in females aged 55–64 years, they significantly increased. The findings emphasize the importance of improving primary and secondary prevention measures, and of diagnostic and logistic improvements, with a focus especially on older females.

## Figures and Tables

**Table 1:** The rates (%) and trends of 28-day acute myocardial infarction case-fatality in the Lithuanian urban residents aged 25–64 years by sex and age during 2000–2023 (JoinPoint regression analysis with 0 JoinPoints).

Years	Males		Females	
	25–54	55–64	25–54	55–64
2000	23.43	32.75	13.17	25.27
2001	22.17	39.25	24.23	16.67
2002	18.30	32.56	16.22	17.89
2003	24.89	33.99	11.26	16.48
2004	30.39	33.74	9.30	25.69
2005	26.57	32.82	8.75	23.15
2006	29.36	37.16	21.23	18.00
2007	36.76	36.24	22.63	21.28
2008	27.99	32.87	18.05	11.58
2009	28.31	37.29	16.56	17.65
2010	22.02	33.62	7.63	19.57
2011	24.56	41.50	15.53	11.49
2012	21.85	36.04	15.23	27.06
2013	18.99	34.78	10.40	24.62
2014	17.34	37.89	14.11	21.54
2015	23.35	38.25	11.23	22.22
2016	27.32	31.58	15.59	32.81
2017	12.64	20.13	21.86	21.25
2018	23.44	26.29	50.00	18.46
2019	12.62	29.86	<0.1 **^#^**	22.97
2020	25.16	32.27	17.62	26.47
2021	37.05	38.76	27.15	51.22
2022	18.72	40.12	24.69	41.86
2023	11.99	32.86	10.08	25.00
Average	23.55	34.28	16.77	23.34
AAPC ^1^ and	−0.9%/yr.,	−0.2%/yr.,	+2.2%/yr.,	+3.0%/yr.,
*p* -value	0.3	0.6	0.2	0.002

^1^ AAPC—average annual percent change; #—very small sample size.

**Table 2:** The rates (%) and trends of 28-day in-hospital acute myocardial infarction case-fatality in the Lithuanian urban residents aged 25–64 years by sex and age during 2000–2023 (JoinPoint regression analysis with 0 JoinPoints).

Years	Males		Females	
	25–54	55–64	25–54	55–64
2000	2.74	7.78	5.72	6.85
2001	5.81	15.03	10.68	7.14
2002	<0.1 **^#^**	11.04	8.82	7.14
2003	3.26	9.24	3.07	3.80
2004	6.08	10.56	<0.1 **^#^**	10.99
2005	3.86	4.92	3.10	4.60
2006	7.32	11.35	8.51	3.53
2007	11.25	12.03	8.62	6.33
2008	7.62	7.64	5.56	2.33
2009	4.75	14.94	5.85	6.67
2010	6.81	12.99	3.97	9.76
2011	5.24	12.03	2.79	6.10
2012	5.64	16.47	<0.1 **^#^**	15.07
2013	6.21	10.45	<0.1 **^#^**	9.26
2014	3.17	14.49	<0.1 **^#^**	7.27
2015	4.53	19.86	5.95	14.29
2016	4.67	11.18	5.80	12.24
2017	2.42	1.65	4.58	7.35
2018	3.53	3.09	9.64	3.64
2019	<0.1 **^#^**	3.27	<0.1 **^#^**	1.72
2020	6.02	5.10	<0.1 **^#^**	1.96
2021	4.91	4.39	<0.1 **^#^**	16.67
2022	2.04	2.83	<0.1 **^#^**	16.67
2023	4.34	5.37	5.31	<0.1 **^#^**
Average	4.68	9.49	4.09	7.56
AAPC ^1^ and	−1.7%/yr.	−2.0%/yr.	−6.7%/yr.	+2.7%/yr.
*p*-value	0.6	0.2	0.2	0.2

^1^ AAPC—average annual percent change; #—very small sample size.

**Table 3:** The trends in age-standardized 28-day acute myocardial infarction case-fatality among the Lithuanian urban residents aged 25–64 years by sex and age during 2000–2023 (JoinPoint regression analysis).

Age Groups	Sex	Joinpoints (Years)	Period 1	APC with 95% CI	Period 2	APC with 95% CI
25–64	Males	2006	2000–2006	2.2 (−5.4; 10.4)	2006–2023	−0.8 (−2.4; 0.7)
	Females	2010	2000–2010	−1.5 (−7.6; 4.9)	2010–2023	5.1 (1.3; 9.1) *
25–54	Males	2021	2000–2021	−0.2 (−2.0; 1.6)	2021–2023	−28.2 (−69.8; 70.7)
	Females	2021	2000–2021	3.0 (−1.7; 8.0)	2021–2023	−26.4 (−90.1; 450.2)
55–64	Males	2019	2000–2019	−0.7 (−1.9; 0.6)	2019–2023	4.1 (−8.7; 18.7)
	Females	2009	2000–2009	−2.5 (−10.7; 6.5)	2009–2023	5.7 (1.7; 9.8) *

APC—annual percent change; 95% CI—95% confidence interval; *—*p* < 0.05.

**Table 4:** The trends in age-standardized in-hospital acute myocardial infarction case-fatality among the Lithuanian urban residents aged 25–64 years by sex and age during 2000–2023 (JoinPoint regression analysis).

Age Group	Sex	Joinpoints (Years)	Period 1	APC with 95% CI	Period 2	APC with 95% CI
25–64	Males	2015	2000–2015	2.2 (−1.5; 6.1)	2015–2023	−14.7 (−24.7; −3.5) *
	Females	2003	2000–2003	−7.9 (−52.9; 80.1)	2003–2023	1.8 (−2.0; 5.8)
25–54	Males	2007	2000–2007	19.1 (−18.1; 73.3)	2007–2023	−6.4 (−15.7; 4.0)
	Females	2021	2000–2021	−8.0 (−18.4; 3.7)	2021–2023	48.1 (−100.0; 467,140.0)
55–64	Males	2015	2000–2015	2.5 (−1.1; 6.3)	2015–2023	−18.3 (−28.5; −6.8) *
	Females	2021	2000–2021	4.0 (−0.2; 8.5)	2021–2023	−40.6 (−96.8; 988.9)

APC—annual percent change; 95% CI—95% confidence interval; *—*p* < 0.05.

## Data Availability

The original contributions presented in this study are included in the article; further inquiries can be directed to the corresponding author.
